# Adoptive Transfer of Dendritic Cells Expressing Fas Ligand Modulates Intestinal Inflammation in a Model of Inflammatory Bowel Disease

**DOI:** 10.4172/2155-9899.1000411

**Published:** 2016-04-29

**Authors:** Edelmarie Rivera de Jesus, Raymond A Isidro, Myrella L Cruz, Harry Marty, Caroline B Appleyard

**Affiliations:** 1 Ponce Health Sciences University-Medical School and Ponce Research Institute, Ponce, PR 00732, USA; 2 Department of Biology, University of Puerto Rico – Ponce Campus, Ponce, PR 00732, USA

**Keywords:** Inflammatory bowel disease, FasL (CD95L), Dendritic cells, Macrophages, Adoptive transfer

## Abstract

**Background:**

Inflammatory bowel diseases (IBD) are chronic relapsing inflammatory conditions of unknown cause and likely result from the loss of immunological tolerance, which leads to over-activation of the gut immune system. Gut macrophages and dendritic cells (DCs) are essential for maintaining tolerance, but can also contribute to the inflammatory response in conditions such as IBD. Current therapies for IBD are limited by high costs and unwanted toxicities and side effects. The possibility of reducing intestinal inflammation with DCs genetically engineered to over-express the apoptosis-inducing FasL (FasL-DCs) has not yet been explored.

**Objective:**

Investigate the immunomodulatory effect of administering FasL-DCs in the rat trinitrobenzene sulfonic acid (TNBS) model of acute colitis.

**Methods:**

Expression of FasL on DCs isolated from the mesenteric lymph nodes (MLNs) of normal and TNBS-colitis rats was determined by flow cytometry. Primary rat bone marrow DCs were transfected with rat FasL plasmid (FasL-DCs) or empty vector (EV-DCs). The effect of these DCs on T cell IFNγ secretion and apoptosis was determined by ELISPOT and flow cytometry for Annexin V, respectively. Rats received FasL-DCs or EV-DCs intraperitoneally 96 and 48 hours prior to colitis induction with TNBS. Colonic T cell and neutrophil infiltration was determined by immunohistochemistry for CD3 and myeloperoxidase activity assay, respectively. Macrophage number and phenotype was measured by double immunofluorescence for CD68 and inducible Nitric Oxide Synthase.

**Results:**

MLN dendritic cells from normal rats expressed more FasL than those from colitic rats. Compared to EV-DCs, FasL-DCs reduced T cell IFNγ secretion and increased T cell apoptosis *in vitro*. Adoptive transfer of FasL-DCs decreased macroscopic and microscopic damage scores and reduced colonic T cells, neutrophils, and proinflammatory macrophages when compared to EV-DC adoptive transfer.

**Conclusion:**

FasL-DCs are effective at treating colonic inflammation in this model of IBD and represent a possible new treatment for patients with IBD.

## Introduction

Inflammatory bowel disease (IBD) is a chronic relapsing and remitting inflammatory condition of the gastrointestinal tract [1] that generally affects the colon, or large intestine, and can be classified as Crohn’s disease or ulcerative colitis. Although these two forms of IBD share common clinical and pathological features, the disease is heterogeneous, with marked differences in clinical presentation, underlying genetic factors, and response to treatment. Like many other chronic inflammatory or autoimmune disorders, the immunopathology of these diseases seems to result from complex interactions between susceptibility genes, the environment (most notably bacteria) and the immune system [2]. Under homeostasis, the intestinal immune system must balance the capacity for mounting protective immune responses to infectious agents (i.e. pathogenic bacteria) with the ability to tolerate the enormous load of antigens and immunostimulatory molecules that constitute the commensal intestinal bacteria (oral and mucosal tolerance) [3]. Therefore, gut inflammation may logically result from a loss of local tolerance.

The intestinal mononuclear phagocyte system, composed of dendritic cells (DCs) and macrophages, is essential for maintaining tolerance. Both macrophages and DCs can serve as antigen presenting cells, having the capacity to initiate and/or regulate intestinal immune responses. Under homeostasis, intestinal macrophages sample luminal contents [4–7] and phagocytose bacteria and other luminal antigens that breach the mucosal barrier, but do not stimulate an overt immune response against these antigens [8–10]. Instead, macrophages transfer antigens to intestinal DCs *via* gap junctions [11], and DCs migrate to mesenteric lymph nodes (MLNs), where they prime naïve T cells and induce their differentiation into regulatory T (Treg) cells. Local production of IL-10 by intestinal macrophages promotes the expansion and maintenance of Treg cells [12,13]. Nevertheless, both intestinal macrophages and DCs propagate the inflammatory response during intestinal inflammation [8–10,14–17], such as occurs in IBD and in animal models of this condition.

Current therapeutic goals mainly focus on decreasing inflammatory cytokine activity by infusing either proinflammatory cytokine-targeting antibodies or anti-inflammatory cytokines, or by using non-specific inhibitors of inflammation, such as corticosteroids or immunosuppressants [18,19]. However, many of these therapies have significant undesirable side effects. Therefore, the identification of a specific molecular and cellular target in the pathogenesis of IBD and new therapeutic agents remains vitally important. Manipulation of DCs or macrophages may open the way towards new therapeutic approaches for IBD. Fas ligand (FasL/CD95L), a type II transmembrane protein that belongs to the tumor necrosis factor family, can induce apoptosis in target cells by binding to its death domain-containing receptor Fas (CD95). In the present study, we show that adoptive transfer of DCs genetically engineered to express FasL, an inducer of apoptosis, can reduce inflammation in a rat model of acute colitis.

## Materials and Methods

### Ethics statement

All experiments involving animals were performed in accordance with institutional, local, and national guidelines and approved by the Ponce Health Sciences University Institutional Animal Care and Use Committee.

### Animal model of colitis

Acute colitis was induced in male Sprague–Dawley rats (250–450 g; Southern Veterinary Service, PR) as previously described [20,21]. The rats were maintained under standard laboratory conditions. Trinitrobenzene sulfonic acid (TNBS; 60 mg/mL) was administered intracolonically after lightly anesthetizing with ether. Control animals were untreated. The rats were weighed daily to monitor weight change as a disease marker, and sacrificed 72 hours after the initial administration of the TNBS by an overdose of pentobarbital. The colon was removed and scored for macroscopic damage using four criteria, as previously described [22]: the presence of adhesions (0, 1, or 2 for none, minor, or major, respectively), diarrhea (0 or 1 for absent or present, respectively), thickness (mm), and ulceration (0 for no damage, with increasing scores depending on extent of ulceration). These were added to give a total macroscopic damage score. After sacrifice, the whole mesenteric lymph node (MLN) chain/layer was identified and removed as previously described [23], trimmed of any fat, cut into pieces, and incubated for 60 min under agitation at 37°C in the presence of 100 U/ml of collagenases type II and VII, and 300 U/ml of hyaluronidase (Sigma). Cells were separated from debris by filtration through a 100 μm cell strainer (BD Bioscience, San Diego, CA) after enzymatic digestion. The flow-through was centrifuged at 1500 rpm for 5 min at 4°C, and the resulting pellet resuspended in RPMI. Dendritic cells and T cells were obtained by FACS.

### Generation, transfection, and adoptive transfer of bone marrow-derived dendritic cells

Bone marrow (BM) cells from male Sprague Dawley rats 8–10 weeks old were isolated as previously described [24]. The BM cells were cultured at a cell density of 2–5 × 10^5^ cells/mL in culture dishes (Falcon, Becton Dickinson Biosciences) or 75 cm^2^ tissue culture flasks (T75) (Corning Inc., Corning, NY, USA). The RPMI 1640 culture medium was supplemented with 20 ng/ml recombinant rat GM-CSF (Sigma Aldrich, St. Louis, MO) and 20 ng/ml recombinant rat IL-4 (Sigma Aldrich, St. Louis, MO), or 20 ng/ml rat GM-CSF (Sigma Aldrich, St. Louis, MO). On day 3 and 6, more growth factors were added. All cells were collected on day 7. Purity was determined by measuring OX62 expression through flow cytometry.

Primary rat BMDCs were transfected with 8 μg of either expression vectors containing rat FasL cDNA (FasL-DCs) or control vectors (EV-DCs) using the Lipofectamine^™^ 2000 Transfection Reagent (Invitrogen, Carlsbad, CA) according to the manufacturer’s protocol. Both vectors were generous gifts from Drs. Li Xiao-Kang and Masayuki Fujino. Each transfection was done in triplicate. Transfection efficiency was determined by measuring FasL expression through flow cytometry.

For adoptive transfer studies, animals received the FasL-DCs or EV-DCs administered intraperitoneally ~3 × 10^7^ cells per rat (based on similar types of studies in arthritis [25]) 96 and 48 hours before the induction of colitis. All animals also received formyl-methionyl-leucyl-phenylalanine (fMLP; 2.5 mM in 6% DMSO at pH 8 intracolonically) 2 hours after the TNBS administration.

### Flow cytometry & fluorescence-activated cell sorting

All flow cytometry analyses and cell sorting experiments were performed using a BD FACSCalibur platform (BD Biosciences) and Cell Quest Pro software (BD Biosciences). DCs and T cells from rat MLN were isolated using mouse anti-rat OX62:PE conjugate (MCA1029PE, AbD Serotec, UK) and mouse anti-rat CD3:FITC conjugate/CD4:RPE conjugate (DC041, AbD Serotec, UK), respectively. Mouse IgG1:RPE (MCA1209PE, AbD Serotec, UK) and mouse IgM: FITC (MCA692F) were used as isotype controls (AbD Serotec, UK). Briefly, the MLN population was suspended in staining buffer (PBS 1x with 0.1% sodium azide and 0.1% FBS) and incubated with 10 μl of antibody per 10^6^ cells for 20 min at 2–8°C in the dark. The cells were washed twice, resuspended in staining buffer and then sorted. The CD3^+^CD4^+^ T, CD3^+^CD4^−^ lymphocytes and OX62^+^ cells (DCs) were gated by size on forward and side scatter. The purity of sorted cells was confirmed by flow cytometry. FasL expression on MLN OX62^+^ cells and on BMDCs transfected with FasL was determined using FITC- and PE-conjugated anti-FasL antibodies (sc-19987; Santa Cruz Biotechnology, Inc., Santa Cruz, CA). Mouse IgM FITC or IgM PE served as isotype controls.

### Annexin V PE Assay

FasL-DCs were co-cultured with CD4^+^ T cells at a ratio of 1:5 in the presence of the bacterial peptide fMLP 10^−7^ M. EV-DCs were used as controls. After 24 hrs of co-culture, an apoptosis assay was performed using Annexin V PE staining kit (BD Bioscience, San Diego, CA) as per the manufacturer’s instructions. To assess the extent of apoptosis, we accounted for both the total number of annexin-positive cells and the intensity of annexin staining by multiplying the percentage of positive cells by the average staining intensity.

### ELISPOT

Solid-phase IFNγ Elispot kits from BD Biosciences (Pharmingen, San Diego, CA) were used to enumerate T cells secreting IFNγ upon co-culture with DCs following fMLP stimulation. In one set of experiments, FasL-DCs or EV-DCs were co-cultured with T cells isolated from MLN of normal rats to examine the immunomodulatory potential of FasL-DCs. The plate was incubated for 24–48 hrs at 37°C in 5% CO_2_. IFNγ production was measured for cell suspensions containing 5 × 10^4^ of either FasL-DCs or EV-DCs alone without treatment, FasL-DCs or EV-DCs treated with fMLP only, or FasL-DCs or EV-DCs treated with fMLP and cocultured with T cells. DCs and T cells were cocultured at a ratio of 1:5, respectively. As a negative control, wells with medium alone were used. Cells were added to individual wells in triplicate, followed by medium with or without antigen (10^−6^M fMLP). The number of spots, which represent IFNγ-producing T cells, were quantified with a dissection microscope or an ELISPOT plate reader (AID). The results were compared with unstimulated cell suspension (no fMLP) as a negative control, or under optimal stimulation with 5 ng/ml of Phorbol Myristate Acetate (Sigma, St. Louis, MO) and 500 ng/ml of Ionomycin (Sigma, St. Louis, MO) as a positive control. Positive controls are not shown in the graph because they produced spots that were deemed too numerous to count. In another set of experiments, the proinflammatory role of MLN DCs was tested *in vitro* by comparing IFN-γ production by either DCs alone, CD4^+^ T cells alone, DCs with CD4^+^T cells, DCs with fMLP, T cells with fMLP or DCs/CD4^+^ T cells with fMLP. The same cell numbers and ratios and reactant concentrations were used in both sets of experiments.

### Western blotting

Forty-eight hours after the gene transfection, the BMDCs were harvested and lysed with Radio Immuno Precipitation Assay buffer consisting of 150 mM sodium chloride 1.0% NP-40, 0.5% sodium deoxycholate, 50 mM Tris, pH 8.0. Proteins were separated by 10% sodium dodecyl sulfate-polyacrylamide gel electrophoresis and transferred to nitrocellulose filters. The filters were blocked with blocking buffer containing 5% skimmed milk and incubated overnight with rabbit anti-FasL antibody (sc-956; Santa Cruz Biotechnology, Inc., Santa Cruz, CA) at 1:500. Horseradish peroxidase-conjugated anti-rabbit IgG (sc-2004; Santa Cruz Biotechnology, Inc.) was added as a secondary antibody at 1:1000 and further incubated for 1 hr. The filters were washed in the blocking buffer and the immune complexes were detected *via* chemiluminescence.

### Histology & immunostaining

Sections of colon were fixed by immersion in 10% buffered formalin, placed in labeled tissue cassettes and processed overnight at RT. Samples were sequentially dehydrated with ascending percentages of ethanol, cleared in xylene, and embedded in paraffin. Four-micron-thick sections were stained with hematoxylin and eosin to determine the extent of inflammatory infiltrate and the appearance of the underlying muscle layers. The resulting slides were analyzed by two blinded observers for disruption of the architecture (0→3; absent→severe), cellular infiltration (0→3; absent→severe), muscle thickening (0→3; absent→severe), presence of crypt abscesses (0 or 1; absent or present), and goblet cell depletion (0 or 1; absent or present) as previously described [22].

Colonic T cell infiltration was determined by staining tissue sections with CD3. Formalin-fixed and paraffin embedded sections obtained from the same histology blocks as outlined above were deparaffinized in xylene and rehydrated through a graded series from ethanol to water. Quenching of endogenous peroxidase was performed by incubating tissue sections with 3% H_2_O_2_ at RT for 15 minutes in a humidified chamber. After washing with PBS (pH 7.4), tissue sections were incubated with 0.25% pepsin at 37°C for 30 minutes to reveal fixed Ag epitopes. Tissue sections were treated with the blocking solution at RT for minutes, followed by incubation with an HRP-conjugated Anti-Rat CD3 (Santa Cruz, CA, USA) at RT for 1 hour. Slides were incubated with DAB staining kit (SK4100; Vector Laboratories, Burlingame, California, USA) for color visualization. Slides were counterstained by incubation with methyl green at 65°C for 3 minutes. Five fields were randomly selected for each section of colon, and the average number of infiltrating T-cells was determined. For negative controls, 10% of normal rat serum was used as the primary antibody.

Double immunofluorescence for CD68 (MCA341B, AbD Serotec) and inducible nitric oxide synthase (iNOS; sc-7271, Santa Cruz) was used to stain colonic sections as previously described [26]. At least 14 high power fields (HPFs) of mucosa were photographed per group. CD68^+^ macrophages and CD68^+^iNOS^+^ proinflammatory macrophages were quantified using the cell counter plugin on ImageJ (NIH, Bethesda, MD).

### Statistical analysis

Data are presented as mean ± standard error of the mean. Statistical significance was set at p values less than 0.05. All statistical analyses were performed using Prism v6.0a (GraphPad Software, Inc., La Jolla, CA, USA). One-way ANOVA with a Holm-Sidak’s multiple comparisons post-hoc test were used for comparing percent weight change across the different time points within the same group. All other comparisons were performed using unpaired, one-sided t test with Welch’s correction.

## Results and Discussion

**Figure 1:**
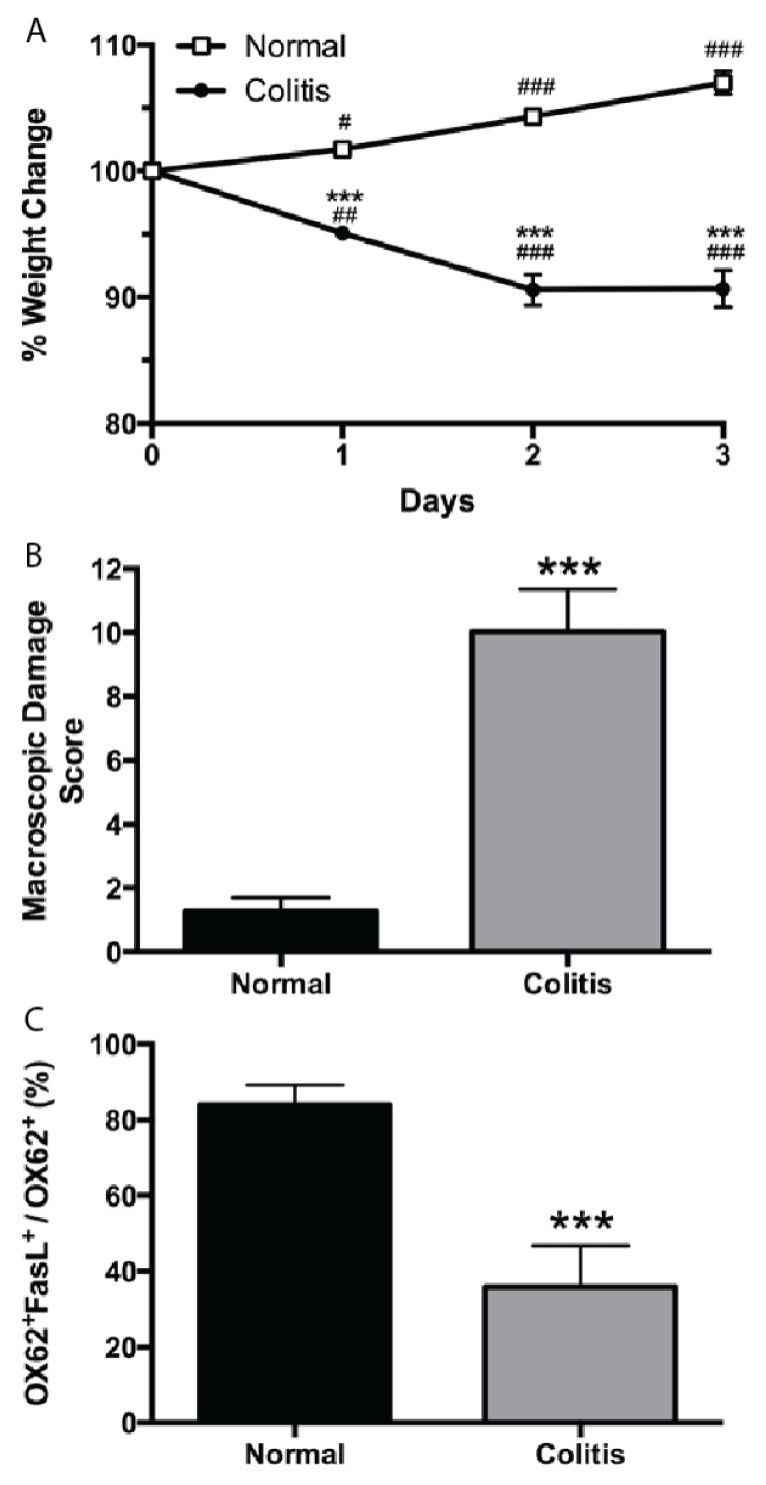
FasL expression on OX62^+^ dendritic cells is decreased in mesenteric lymph nodes (MLNs) from rats with acute colitis. **A.** weight change in normal and acute colitis rats expressed as percent of initial weight on day of colitis-induction (day 0). **B.** macroscopic damage scores for normal rats and rats with acute colitis. C, percentage of OX62^+^ MLN cells that are OX62^+^FasL^+^ in normal and colitic rats. Representative flow cytometry blots for panel C are shown in Supplemental Figure 1. n=4 rats per group. ^***^ p<0.001 vs normal group; ^#^ p<0.05, ^##^ p<0.01, ^###^ p<0.001 vs day 0 of same group.

We first asked whether DCs in colitic rats were functionally different from those in normal control rats. As expected, rats receiving TNBS-induced colitis lost more weight (Figure 1A) and had higher macroscopic damage scores (Figure 1B) than normal controls. Analyzing FasL expression by flow cytometry on MLN-DCs from normal and colitic rats revealed a marked reduction in the expression of FasL on MLN-DCs from colitic rats when compared to MLN-DCs from normal controls (p<0.001; Figure 1C, and Supplemental Figure 1). These results suggest that MLN-DCs promote inflammation in this model of acute colitis. To confirm the proinflammatory role of DCs, we examined IFNγ production by MLN-DCs, MLN-CD4^+^ T cells, and cocultures of MLN DCs and CD4^+^ T cells in the presence and absence of the bacterial peptide fMLP. Few DCs or T cells produced IFNγ when cultured alone and exposed to fMLP. However, the number of IFNγ- producing cells was substantially increased when DCs and T cells were cocultured in the presence of fMLP (data not shown).

**Figure 2:**
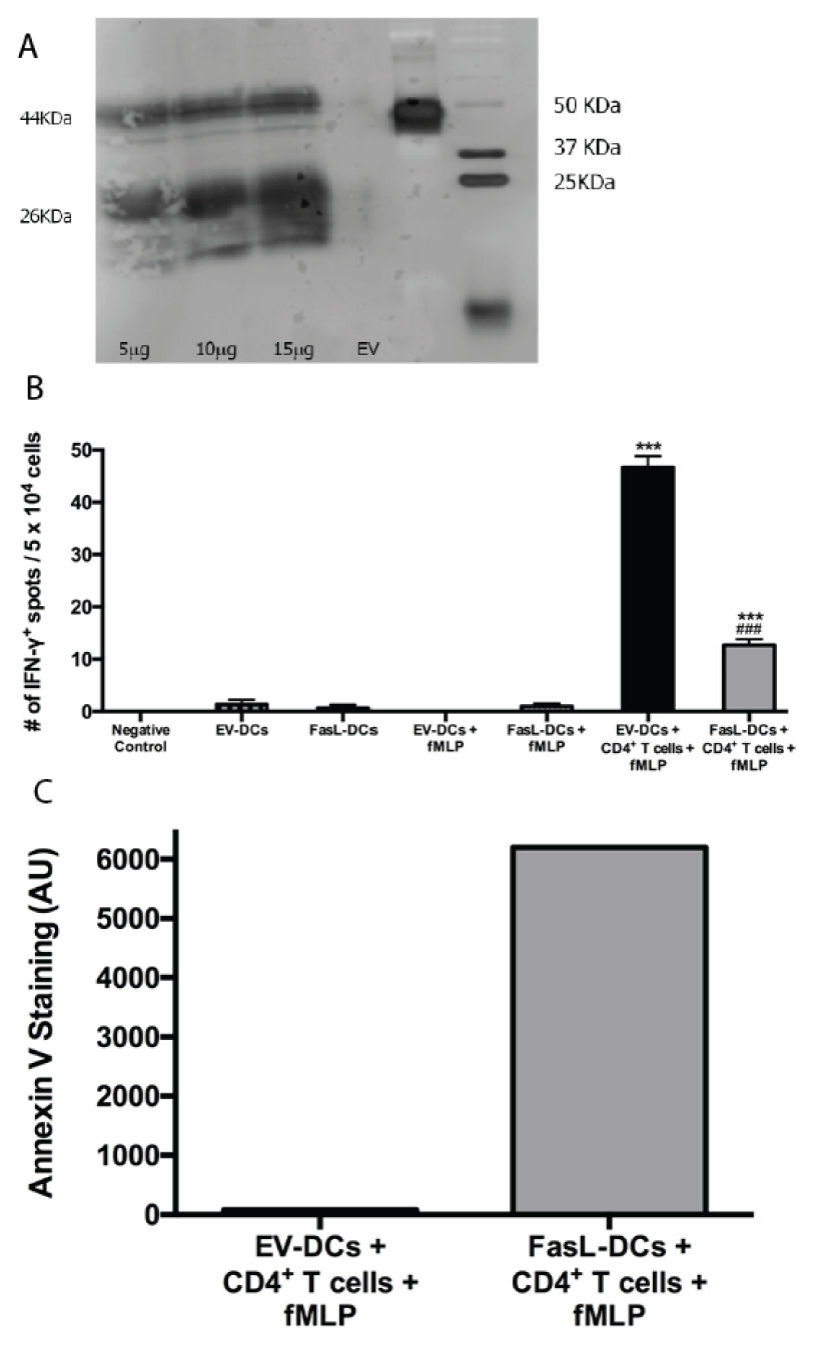
Dendritic cells expressing FasL (FasL-DCs) reduce interferon-γ production and induce apoptosis *in vitro*. **A.** Western blot to determine the expression of FasL by bone marrow-derived dendritic cells transfected with 5, 10, or 15 μg of FasL-coding vector or 15 μg of empty vector (EV-DCs). Membrane-bound (44 KDa) and soluble (26 KDa) forms of FasL were observed. **B.** ELISPOT to determine IFN-γ production by EV-DCs and FasL-DCs cultured alone with or without treatment with formyl-methionyl-leucyl-phenylalanine (fMLP) or cocultured with CD4^+^ T cells and treated with fMLP. n=3 per group. ^***^ p<0.001 vs negative control, ^###^ p<0.001 vs EV-DCs and CD4^+^ T cell coculture. C. Flow cytometry for annexin-V staining (AU) in cocultures of T cells and EV-DCs or FasL-DCs that were treated with fMLP. Representative flow cytometry blots for panel C are shown in Supplemental Figure 2.

Apart from inducing apoptosis in target cells upon ligating the Fas receptor on their cell membranes, FasL appears to contribute to the resolution of inflammation as evidenced by persistent and severe colitis in FasL knockout mice [27]. Additionally, T cells upregulate Fas expression upon interaction with dendritic cells and, thus, become sensitive to Fas/FasL-induced apoptosis [28,29]. We therefore hypothesized that overexpressing FasL on DCs *via* transfection could impart immunomodulatory characteristics on the transfected DCs. Transfection efficiency was determined to be 5.53%, 11.77%, and 19.97% by flow cytometry for cells transfected with 5 μg, 10 μg, and 15 μg of FasL-coding vector, respectively. FasL expression was confirmed by western blotting (Figure 2A). Next, we analyzed IFNγ production in FasL-DCs or EV-DCs that were cultured alone without treatment, cultured alone with fMLP treatment, or cocultured with CD4^+^ T cells and treated with fMLP (Figure 2B). FasL-DC and T cell cocultures contained significantly fewer IFNγ-producing cells than cocultures of EV-DCs and T cells (p<0.001). Few DCs cultured alone produced IFNγ regardless of transfection and treatment with fMLP. Furthermore, Annexin V-positivity, an indicator of apoptosis, was much greater in FasL-DCs/CD4^+^ T cell cocultures than in cocultures containing EV-DCs (Figure 2C, and Supplemental Figure 2). Even though mature DCs have been shown to express Fas, studies have demonstrated that they have an increased expression of anti-apoptotic molecules such as c-FLIP and Bcl-xL, probably leading to their protection from apoptosis [30–32]. Therefore, FasL-DCs attenuate T cell inflammatory cytokine production in vitro likely by inducing T cell apoptosis.

**Figure 3:**
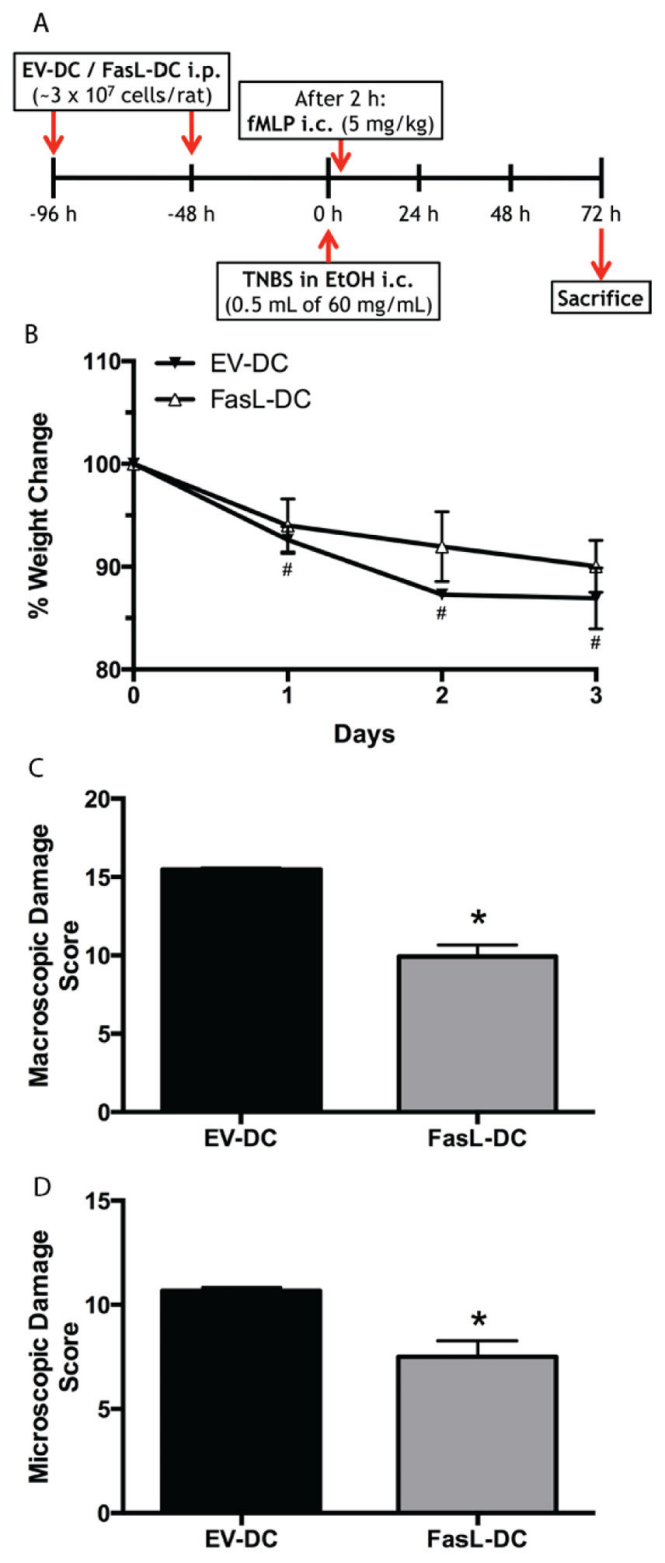
Adoptive transfer of dendritic cells expressing FasL (FasL-DCs) attenuates macroscopic and microscopic damage in an acute colitis model. **A.** timeline for adoptive transfer and acute colitis induction protocol. **B.** Weight change in colitic rats that received either FasL-DCs or dendritic cells transfected with an empty vector (EV-DCs), expressed as percent of the initial weight on the day of colitis induction (day 0). **C.** Macroscopic damage scores for rats treated with FasL-DCs or EV-DCs. **D.** Microscopic damage scores for rats treated with FasL-DCs or EV-DCs. n=2 rats per group. ^#^p<0.05 vs day 0 of same group; ^*^p<0.05 vs EV-DC group.

To investigate the *in vivo* immunomodulatory potential of FasL-DCs, we employed an acute colitis animal model of IBD in which we adoptively transferred FasL-DCs or EV-DCs to rats prior to colitis induction (Figure 3A). Rats that received the EV-DCs instead of the FasL-DCs weighed significantly less than their original weight by the end of the study (p<0.05; Figure 3B). In contrast, weights after colitis induction were not significantly different from original for rats receiving FasL-DCs. Furthermore, adoptive transfer of FasL-DCs significantly decreased colonic damage both macroscopically and microscopically (p<0.05; Figures 3C and 3D). Although previous studies have shown the immunomodulatory potential of regulatory dendritic cells in animal models of colitis [33–37], ours is the first study that shows that expression of FasL on BMDCs resulting from genetic manipulation is sufficient to attenuate colonic inflammation in a model of acute colitis.

**Figure 4:**
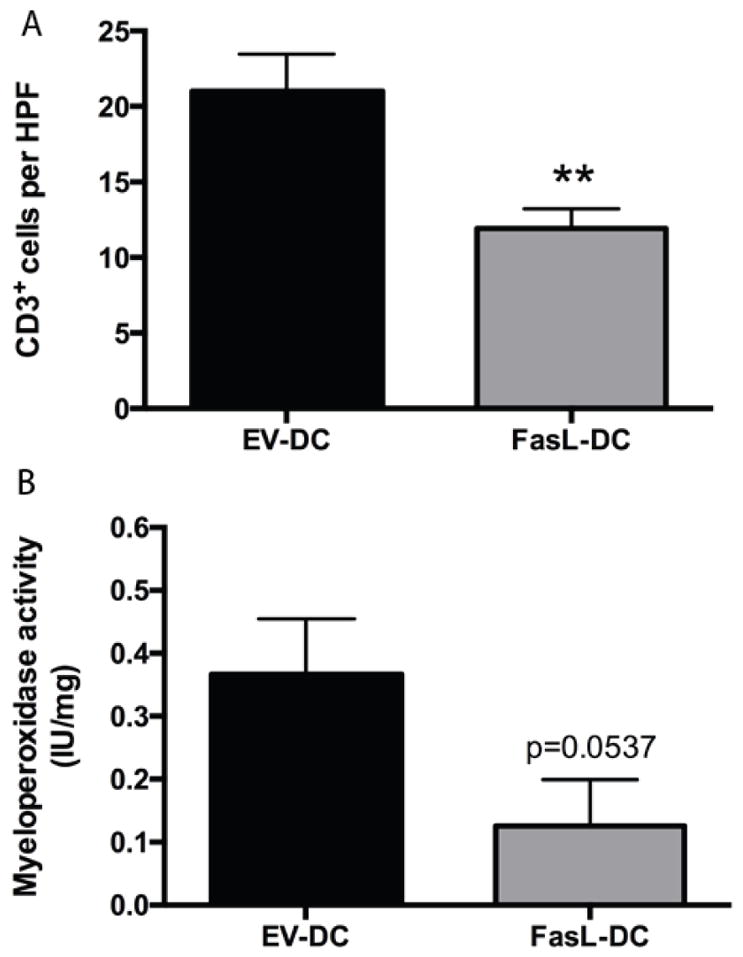
Adoptive transfer of dendritic cells expressing FasL (FasL-DCs) reduces colonic T cell and neutrophil infiltration. **A.** quantification of CD3^+^ cells per high-power field (HPF, 400×) of colonic tissue from rats treated with FasL-DCs or dendritic cells transfected with an empty vector (EV-DCs). Tissues were stained by immunohistochemistry for CD3. n=30 HPFs per group. **B.** Myeloperoxidase activity (IU/mg) in colonic tissue from EV-DC-and FasL-DC-treated rats. n=2 per group. ^**^ p<0.01 vs EV-DC group.

**Figure 5:**
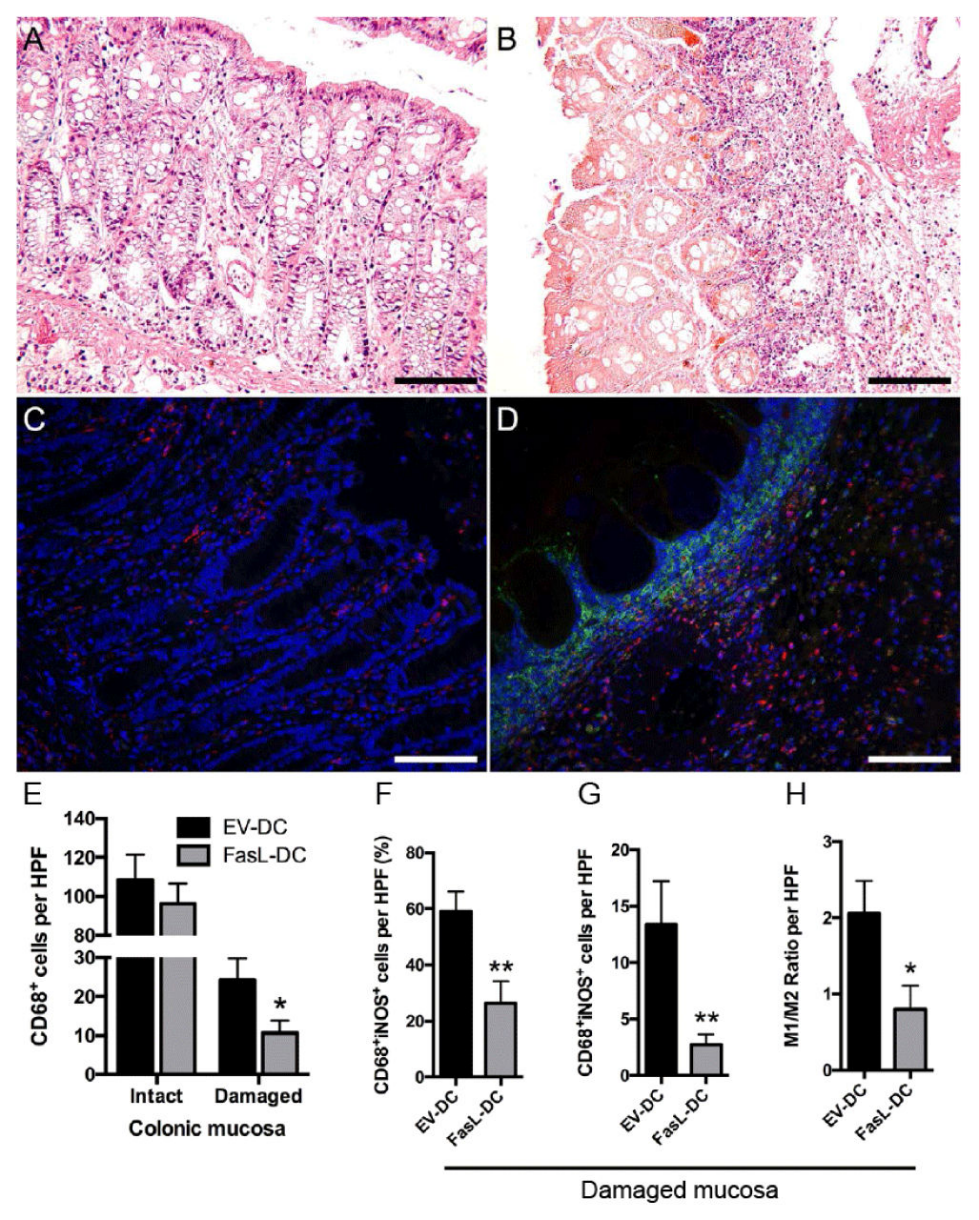
Adoptive transfer of dendritic cells expressing FasL (FasL-DCs) decreases the proportion of colonic proinflammatory macrophages. Representative micrographs of hematoxylin and eosin staining depicting the histology of intact (**A**) and damaged (**B**) colonic mucosa from colitic rats (400×). Representative micrographs of double immunofluorescent staining for CD68 (red) and inducible nitric oxide synthase (iNOS, green) of intact (**C**) and damaged (**D**) colonic mucosa from colitic rats (400×). (**E**) Number of CD68^+^ cells per high-power field (HPF, 400×) of colonic tissue from rats treated with FasL-DCs or dendritic cells transfected with an empty vector (EV-DCs). (**F**) quantification of CD68^+^iNOS^+^ cells per HPF of colonic tissue from rats treated with FasL-DCs or EV-DCs, expressed as a percentage of total CD68^+^ cells. (**G**) Number of CD68^+^iNOS^+^ cells per HPF of colonic tissue from rats treated with FasL-DCs or EV-DCs. (**H**) M1/M2 ratio (calculated CD68^+^iNOS^+^/CD68^+^iNOS^−^) per HPF of colonic tissue from rats treated with FasL-DCs or EV-DCs. n=14–15 HPFs for intact mucosa, 15–17 HPFs for damaged mucosa. ^*^ p<0.05, ^**^ p<0.01 vs EV-DC group. Scale bars = 100 μm.

To investigate the mechanism by which FasL-DCs ameliorate inflammation in this acute colitis model, we examined the effect that FasL-DC adoptive transfer had on T cell, neutrophil, and macrophage infiltration. CD3^+^ T cells were significantly reduced in colonic mucosa from FasL-DC rats when compared to EV-DC rats (p<0.01; Figure 4A). Colonic tissue from FasL-DCs rats demonstrated less MPO activity, an indicator for neutrophils and other myeloid cell infiltration, than colonic tissue from EV-DC rats (p=0.0537; Figure 4B). Upon examining macrophage infiltration, we found that areas of histologically intact mucosa contained more macrophages than areas of damaged mucosa (Figure 5). Damaged mucosa was characterized by regions of marked eosinophilia, indicative of cell death and likely the result of necrosis, with underlying massive inflammatory infiltrates (Figure 5B). Notably, these inflammatory infiltrates were strongly positive for iNOS immunofluorescent staining (Figure 5D). Although total macrophage numbers between EV-DC and FasL-DC rats did not differ significantly in areas of intact mucosa, FasL-DC rats contained less proinflammatory macrophages (p<0.01) and total macrophages (p<0.05) in areas of damaged mucosa than EV-DC rats (Figures 5E–5H). T cells, neutrophils, and macrophages, among other cells, express Fas. T cells have long been known to undergo apoptosis upon Fas ligation, and neutrophils have recently been shown to be regulated by Fas ligation *in vivo* [38]. FLIP expression by macrophages is thought to account for the resistance to Fas ligation exhibited by these cells [39], yet pathogen-associated molecular patterns (PAMPs) can sensitize macrophages to Fas-mediated apoptosis and lead to a proinflammatory state in these cells upon Fas ligation [40]. In contrast to proinflammatory macrophages, resident intestinal macrophages are hyporesponsive to PAMPs despite potent antibacterial activity. Therefore, resident and proinflammatory intestinal macrophages might respond differently to Fas ligation. Interestingly, we observed that, when compared to EV-DC treatment, treating rats with FasL-DCs decreased total colonic macrophage numbers in damaged, but not intact, areas of colon and reduced proinflammatory colonic macrophages. This suggests that FasL-DCs modulate proinflammatory macrophages by inducing apoptosis *via* Fas ligation. Future studies should aim to better understand the mechanism by which FasL-DCs reduce colonic inflammation. Furthermore, the possibility of transfecting induced regulatory DCs to express FasL should be investigated as a way of generating more potent disease-modifying DCs.

In conclusion, we have shown that FasL-DCs are effective at treating colonic inflammation in this animal model of IBD. The use of FasL-DCs should be investigated further as a potential therapy for patients with IBD.

## Supplementary Material

Supplementary file

## Abbreviations

Bcl-xL: B-cell Lymphoma-extra Large
BM: Bone Marrow
BMDC: Bone Marrow-derived Dendritic Cell
c-FLIP: cellular Fas-associated death domain-like IL-1 beta-converting enzyme (FLICE)-inhibitory protein
CD#: Cluster of Differentiation #
cDNA: Complementary Deoxyribonucleic Acid
DAB: Diaminobenzidine
DC: Dendritic Cell
ELISPOT: Enzyme-Linked Immunospot Assay
EV-DC: Bone-marrow derived dendritic cell transfected with an empty vector
FACS: Fluorescence-activated Cell Sorting
FasL: Fas Ligand (CD95L)
FasL-DC: Bone-marrow derived dendritic cell transfected to over-express FasL
FBS: Fetal Bovine Serum
FITC: Fluorescein Isothiocyanate
fMLP: formyl-methionyl-leucyl-phenylalanine
G-CSF: Granulocyte Colony-stimulating Factor
GM-CSF: Granulocyte Monocyte Colony-stimulating Factor, also known as CSF2
HPF: High-power Field
IBD: Inflammatory Bowel Disease
IFNγ: Interferon-gamma
IgG1: Immunoglobulin G subclass 1
IgM: Immunoglobulin M
IL-4: Interleukin 4
iNOS: inducible Nitric Oxide Synthase
MLN: Mesenteric Lymph Node
OX62: anti-integrin alpha E antibody
PAMP: Pathogen-associated Molecular Pattern
PBS: Phosphate Buffered Saline
PE: Phycoerythrin
PMA: Phorbol Myristate Acetate
RT: Room Temperature
TNBS: Trinitrobenzene Sulfonic Acid
Treg: Regulatory T cell
