# Supplementary material for: Adoptive Transfer of Dendritic Cells Expressing Fas Ligand Modulates Intestinal Inflammation in a Model of Inflammatory Bowel Disease
Source: J Clin Cell Immunol. Author manuscript; Available in PMC 2016 Jun 3. (PMC4892183; doi:10.4172/2155-9899.1000411)
Supplement: Supplementary file [file NIHMS787796-supplement-Supplementary_file.pdf]

A.

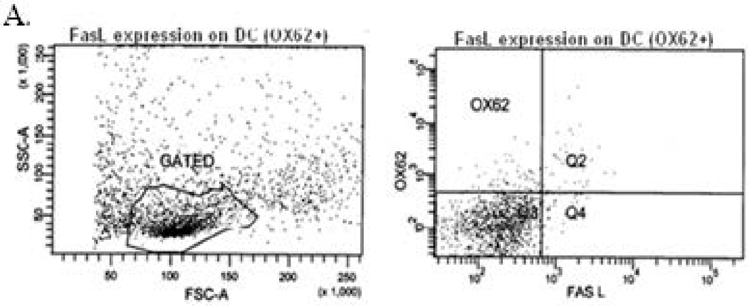

Experiment Name: FasL expression on DC (OX62+)
Tube Name: OX62/FAS L
Record Date: Aug 12, 2008 1:55:43 PM

Population	#Events	%Parent	FITC-A Mean	OX62 PE-A Mean
All Events	4,259	####	2,211	1,255
OX62	175	4.1	409	923
Q2	1,060	24.9	8,009	4,595
Q3	2,775	65.2	208	89
Q4	249	5.8	1,119	270
GATED	2,323	54.6	232	160

B.

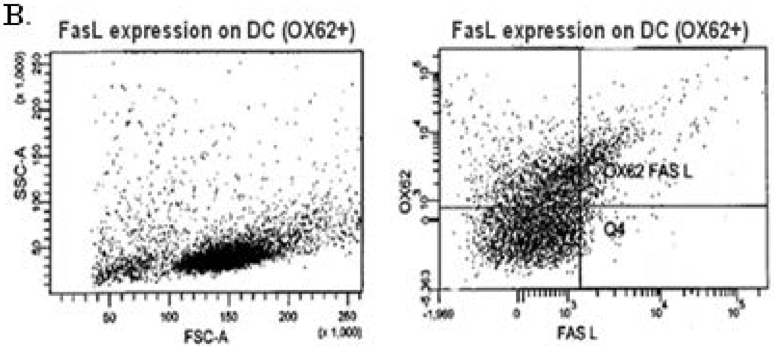

Experiment Name: FasL expression on DC (OX62+)
Tube Name: OX62/FAS L
Record Date: Sep 10, 2008 8:43:14 AM

Population	#Events	%Parent	FAS L FITC-A Mean	OX62 PE-A Mean
All Events	10,000	####	953	1,723
Q1	3,370	33.7	440	3,161
OX62 FA...	1,339	13.4	4,869	7,668
Q3	5,120	51.2	234	-710
Q4	171	1.7	1,953	-336

Supplemental Figure 1. Representative expression of FasL on MLN OX62$^+$ cells. A, FasL expression on OX62$^+$ cells from a normal rat. **B,** FasL expression on OX62$^+$ cells from a colitis rat.

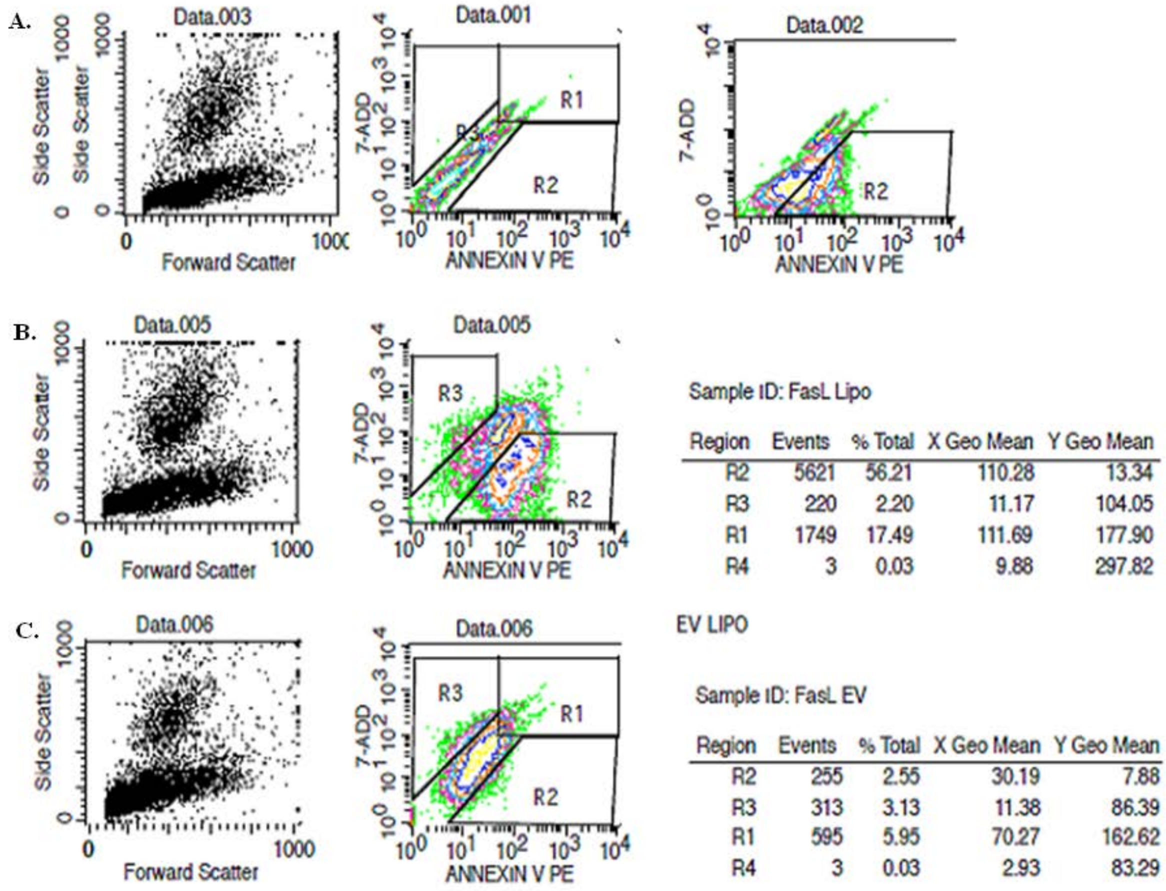

Sample ID: FasL Lipo

Region	Events	% Total	X Geo Mean	Y Geo Mean
R2	5621	56.21	110.28	13.34
R3	220	2.20	11.17	104.05
R1	1749	17.49	111.69	177.90
R4	3	0.03	9.88	297.82

EV LIPO

Sample ID: FasL EV

Region	Events	% Total	X Geo Mean	Y Geo Mean
R2	255	2.55	30.19	7.88
R3	313	3.13	11.38	86.39
R1	595	5.95	70.27	162.62
R4	3	0.03	2.93	83.29

Supplemental Figure 2. Representative Annexin V staining. A, Control (non-transfected cells). **B,** FasL-DCs cocultured with T cells and treated with fMLP. **C,** EV-DCs cocultured with T cells and treated with fMLP. 5,527% more apoptosis was found in FasL-DCs cocultured with $CD4^+$ T cells in presence of fMLP in comparison with EV-DCs cocultured with $CD4^+$ T in the presence of fMLP.
